# “The Insane Asylum” for “Gastric Diseases”

**Published:** 1910-11

**Authors:** George M. Gould

**Affiliations:** Ithaca, N. Y. The Sentinels, Cayuga Heights


### "The Insane Asylum" for "Gastric Diseases"

BY GEORGE M. GOULD, M.D.
Ithaca, N. Y.

ON March 21, 1910, a woman, 26 years of age, was sent to me by a physician from a distant part of the state with a history during the last 6 or 8 years of severe headaches and "bilious attacks," vomiting seizures, fainting, much stomachal trouble, constipation, dysmenorrhea, "nervousness," denutrition, and the like.

The woman's mind was as thoroughly morbid as her body, and she seemed destined, indeed, for some asylum for the insane. The patient, and also her physicians (prior to the last one who sent her to me) had diagnosticated her case as "gastric disease." I found her wearing, from a great ophthalmological professor in a distant city, the following glasses;

R.+sph. 0.25—cyl. 2.50 ax. 180°
L.+sph. 0.25—cyl. 2.50 ax. 180°

Mydriasis, however, demonstrated the following error:

R.—sph. 0.25+cyl. 2.25 ax. 90°
L.+cyl. 2.00 ax. 90°

It was an amazing, utterly inexplainable thing, that professor's prescription, but such things are happening every day in ophthalmology. On June 9, 1910, I received a letter from Dr. C. who had referred the woman to me saying:

I never saw such wonderful results as in the case of Miss L. She has gained twenty pounds since March 19, 1910. She sleeps like a top, eats anything she wishes, and has never taken a single laxative since she has worn your glasses. She now menstruates regularly, whereas up to the time she went to you several months usually passed between periods. She looks and acts like a different girl, and as she expressed it, "I felt as though I should end up in an insane asylum, if I did not get to feel better." She has had one or two headaches after long hours of embroidering at night. I write this for I do appreciate so much what you have done for the girl.

Why did this woman get well in a jiffy when given correct lenses for her frightful astigmatism? Because one-tenth of the amount of her astigmatism may produce the most intense suffering, life long, and may beget diseases seemingly organic, of many organs far-distant from the eyes; because her old glasses artificially created some ten times more astigmatism than she had naturally; because, especially because, chiefly because, she was not over 40 years of age; because she was only 26 years of age; because her kind general physician had seen so many cures in just such cases, of "gastric disease," by means of glasses; because this physician had seen incorrect lenses fail to cure; because, instead of making money out of the poor girl, she spent money for her to pay her heavy traveling expenses; because she followed up the subsequent history and demonstrated the action of cause and effect. The treatment of patients without adequate history-taking, for diseases of unknown etiology, after caricature diagnosis, by blind therapeutics, the subsequent history and cure unsuspected,—such things do not constitute true Medicine, but the pseudo-medicine which awaits the on-coming revolution of an aroused public sentiment. How much better it would be if an aroused professional sentiment should prevent the needless overturn!

THE SENTINELS, CAYUGA HEIGHTS.

